# Radiomic Profiling of Orthotopic Mouse Models of Glioblastoma Reveals Histopathological Correlations Associated with Tumour Response to Ionising Radiation

**DOI:** 10.3390/cancers17081258

**Published:** 2025-04-08

**Authors:** Nicoleta Baxan, Richard Perryman, Maria V. Chatziathanasiadou, Nelofer Syed

**Affiliations:** 1 John Fulcher Neuro-Oncology Laboratory, Department of Brain Sciences, Faculty of Medicine, Imperial College London, London W12 0NN, UK; r.perryman13@imperial.ac.uk (R.P.); m.chatziathanasiadou@imperial.ac.uk (M.V.C.); 2 Biological Imaging Centre, Hammersmith Campus, Imperial College London, London W12 0NN, UK

**Keywords:** glioblastoma, radiomics, histopathology, MRI

## Abstract

Background: Glioblastoma (GB) is a particularly malignant brain tumour which carries a poor prognosis and presents limited treatment options. MRI is standard practice for differential diagnosis at initial presentation of GB and can assist in both treatment planning and response assessment. MRI radiomics allows for discerning GB features of clinical importance that are not evident by visual analysis, augmenting the morphological and functional tumour characterisation beyond traditional imaging techniques. Given that radiotherapy is part of the standard of care for GB patients, establishing a platform for phenotyping radiation treatment responses using non-invasive methods is of high relevance. Methods: In this study, we modelled the responses to ionising radiation across four orthotopic mouse models of GB using diffusion and perfusion radiomics. We have identified the optimal set of radiomic features that reflect tumour cellularity, microvascularity, and blood flow changes brought about by radiation treatment in these murine orthotopic models of GB, and directly compared them with endpoint histopathological analysis. Results: We showed that the selected radiomic features can quantify textural information and pixel interrelationships of tumour response to radiation therapy, revealing subtle image patterns that may reflect intra-tumoural spatial heterogeneity. When compared to GB patients, similarities in selected radiomic features were noted between orthotopic murine tumours and non-enhancing central tumour areas in patients, along with several discrepancies in tumour cellularity and vascularization, denoted by distinct grey level intensities and nonuniformity metrics. Conclusion: As the field evolves, radiomic profiling of GB may enhance the evaluation of targeted therapeutic strategies, accelerate the development of new therapies, and act as a potential virtual biopsy tool.

## Simple Summary

Glioblastoma (GB) is an aggressive form of brain cancer that is usually rapidly fatal. MRI plays an important role in the evaluation of GB, both at initial diagnosis and follow up after radiotherapy. Radiomics is an advanced technique that uses computer algorithms to extract and analyse a wealth of detailed information from radiological scans, going beyond what the human eye can perceive. Radiomic profiling of GB using MRI can yield characteristics related to the tumour’s morphological and functional responses post treatment, turning standard medical images into a rich source of quantitative data. In this study, we propose a radiomic framework based on MRI diffusion and perfusion metrics to model responses to ionising radiation across several orthotopic mouse models of GB. Our findings provided valuable and translatable insights into radiation treatment responses and evaluated the suitability of orthotopic mouse models of GB as representatives of human GB. This may improve the pre-clinical evaluation of targeted therapeutic strategies, accelerate the development of new treatments, and serve as a potential non-invasive biopsy alternative.

## 1. Introduction

Glioblastoma (GB) is a highly invasive brain tumour with poor prognosis and limited treatment options. The current standard of care consists of maximal safe tumour resection followed by radiation and temozolomide chemotherapy [1,2]. MRI provides excellent soft tissue contrast and can reveal details about water diffusivity, cellularity [3], blood flow [4], and tumour perfusion [5], making it standard practice for differential diagnosis at initial presentation of GB, assisting in both treatment planning and response monitoring. Nonetheless, such evaluations can prove to be challenging. Quantitative MRI, through the use of radiomics [6], can provide a better tool for characterising tissue properties at the microscale, capturing novel and valuable information about structural and vascular characteristics of tumour behaviour before and after treatment.

Several studies have explored the use of MRI radiomics in GB, focusing on various aspects of diagnosis, prognosis, and treatment monitoring. For instance, an integrative radiomic model was reported showing differentiation in GB from metastases by utilising a combination of contrast-enhanced T1- and T2-weighted images, along with diffusion-weighted and apparent diffusion coefficient (ADC) mapping [7]. Improved prognostic capabilities of newly diagnosed GB patients were outlined through the use of conventional diffusion-, and perfusion-weighted MR radiomics, demonstrating improved performance over clinical predictors alone [8]. In addition, several studies have shown promise in differentiating low-grade gliomas from high-grade ones using textural radiomic analysis [9,10], while others have shown potential in predicting treatment outcomes and assessing pseudoprogression in high-grade gliomas [11,12]. Tumour radiomics has also recently emerged as a tool to predict adult high-grade glioma subtypes, showing the ability to classify gliomas into two distinct molecular subgroups, IDH-wildtype and IDH-mutant, in patients [13]. Radiomics features derived from diffusion ADC and cerebral blood volume mapping may also assist in differentiating tumour recurrence from radiation necrosis, a prevalent challenge encountered in the management of GB following radiotherapy [14,15].

Applying MRI radiomic analysis to experimental mouse models of GB provides an opportunity to explore underlying mechanisms of disease and treatment, with a higher level of rigour and detail than typically found in clinical settings. As such, animal models provide a controlled environment for evaluating radiotherapy-induced tissue damage, enabling direct histopathological confirmation.

In this study, we sought to establish a radiomic framework for modelling responses to ionising radiation (IR) across four orthotopic mouse models of GB (three syngeneic, GL261 [16], CT-2A [17], and NPE-IE [18], and one patient-derived xenograft, GBM96 [19]), by incorporating quantitative MRI protocols based on diffusion (ADC) and perfusion (CBF) mapping. By employing these quantitative MRI techniques, more reproducible analysis can be achieved, which may result in more consistent outcomes across different scanners and institutions [20,21]. Given that quantitative MRI methods are expected to remain stable across different magnetic field strengths and are less affected by technical factors such as b-value selection, improved consistency can also be attained when translating preclinical quantitative MRI-derived radiomic studies to patient settings.

We propose evaluating the feasibility of using ADC and CBF radiomics to improve the classification of radiation treatment responses in the presented GB orthotopic mouse models. We aimed to determine the optimal set of radiomic features that could act as potential imaging markers associated with endpoint histopathology to evaluate tumour cellularity and vessel integrity post-radiation. We examined a set of twenty adult GB patients, including individuals with and without O6-methylguanine-DNA methyltransferase (MGMT) methylation [22], to assess the similarities and distinctions between orthotopic murine tumours and the enhancing/non-enhancing central tumour regions in patients. We noted that GL261 and CT-2A displayed an increased tumour cellular density and reduced vascularisation in comparison to GB patients, while the NPE-IE mouse model revealed hyperperfused areas comparable to those seen clinically in patients.

The findings presented here create a distinctive MRI radiomics platform for phenotyping IR treatment responses in mouse models of GB. This is valuable for assessing the efficacy of therapeutic strategies and evaluating the suitability of different orthotopic mouse models of GB as representative pre-clinical models of human GB. The establishment of radiomic biomarkers for tissue damage may also enable non-invasive “virtual biopsies” which can assist in the diagnosis and follow up of brain tumour patients.

## 2. Materials and Methods

All animal experiments were conducted in accordance with the UK Home Office Animals (Scientific Procedures) Act 1986, and conform to the guidelines from Directive 2010/63/EU of the European Parliament on the protection of animals used for scientific purposes or the NIH Guide for the Care and Use of Laboratory Animals. All animal procedures were reviewed and approved by the Imperial College Animal Welfare and Ethical Review Body. The authors complied with the ARRIVE guidelines.

### 2.1. Establishment of Orthotopic Glioma Tumours

Orthotopic models of glioma, including one patient-derived xenograft model, were established as follows. Eight- to ten-week-old female C57BL/6J or athymic nude mice, purchased from Charles River, were anaesthetised using isoflurane (1–5%), and given pre-operative analgesia of buprenorphine (0.1 mg/kg) and carprofen (5 mg/kg). The scalp was prepared for surgery by shaving and then the application of depilatory cream, followed by sterilisation with iodine. The mice were then moved to a stereotaxic frame, and a single incision was made along the midline of the scalp to expose the skull. A 10 µL Hamilton syringe (Hamilton Company, Reno, NV, USA) with a glass needle was aligned to the following coordinates relative to the bregma: anteroposterior +1 mm, mediolateral +2 mm. A borehole was then drilled to expose the meninges, and the needle was loaded with either GL261, CT-2A, NPE-IE, or primary GB cells. The needle was lowered through the borehole to a depth of −3 mm relative to the bregma to reach the right striatum, and 2 µL of PBS containing 5 × 10^4^ cells were delivered using a micropump injector over 2 min. The needle was then retracted, the skin was sutured, and the mice were given water containing carprofen (33 ug/mL) for post-operative pain management. Tumours were allowed to establish for at least 3 days post-surgery prior to imaging and treatment. The mice were randomly divided into two groups: control (saline-treated) and IR-treated mice.

This study utilised data from multiple projects to minimise the total number of animals used, adhering to the principles of the 3 Rs (refinement and reduction). A minimum of 4 animals was employed for each group, with additional subjects included when available. Animal imaging was conducted in compliance with local project licence regulations. The NPE-IE model, being more recently acquired, was used to validate data from GL261 and CT-2A models using these cells, and, as such, had multiple studies running concurrently with more saline-treated mice available in our study. The GBM96 model was still being actively developed in-house, resulting in a smaller number of subjects available in the IR-treated group. Data from healthy tissue (measured on the contralateral side) were combined across C57BL/6 and nude athymic mice, as no significant differences were observed between strains. This approach allowed us to optimise the use of available resources while minimising animal usage.

### 2.2. Molecular Profile of Cell Lines Used in This Study

The GL261 and CT-2A models are chemically induced mouse glioma models originating from C57BL/6 mice. GL261 has multiple whole chromosomal changes, alongside a mutation in Kras [23]. CT-2A has alterations in specific chromosomal regions, including a loss of Cdkn2a/b, paired with a point mutation in Nras [24]. Both models display alterations in Nf1 and Trp53, which are often mutated in GB. Whilst RAS mutations are rare in GB, alterations in RAS signalling are commonly observed due to mutations in NF1 and other upstream receptor tyrosine kinases [25]. The NPE-IE model is a genetically engineered mouse glioma cell line created from neural stem cells derived from C57BL/6 mice, which have been genetically modified with Nf1, Pten, and EGFRvIII mutations, and subsequently passaged in C57BL/6 mouse brains to create an immunoevasive phenotype, which recapitulates human GB more closely [18]. GBM96 is a primary GB patient-derived xenograft (PDX) model with mutations in TP53, PIK3CA, and ERBB2, as determined by RNA sequencing. There is no evidence of mutations in IDH1/2 or ATRX, or other genes known to be mutated in GB such as PTEN, NF1, or CDKN2A/B. Histopathology showed an amplification of EGFR on the original tissue, and RNA sequencing data indicate that this is retained in the cell line derived from the same patient. 1p/19q was not measured clinically, but RNA sequencing data also indicate that there may be a 1p/19q codeletion, although this has not been independently validated. The TERT promoter mutation was not assessed clinically, and mutations in the TERT promoter were not measurable by RNA sequencing, and so were undetermined.

### 2.3. Radiation Treatment

Tumour-bearing mice were subjected to radiotherapy as follows. The mice were anaesthetised using a combination of ketamine (50 mg/kg) and medetomidine (0.5 mg/kg). Once anaesthesia had set in, the mice were placed on plastic supports on their backs, then onto lead shielding placed within a radiation chamber, leaving their heads exposed. The entire mouse head was then exposed to 4–8 Gy gamma irradiation from a caesium-137 source. The sedative effects of anaesthesia were then reversed using atipamezole (2.5 mg/kg), and the mice were allowed to recover in a warming chamber.

### 2.4. MRI Experiment

For the MRI procedure, the mice were initially anaesthetised in an induction chamber using a mixture of 3% isoflurane and oxygen. Anaesthesia was maintained during MRI scanning by administering 2% isoflurane through a nose cone. The mice were positioned prone on a dedicated mouse bed. To maintain the mouse’s body temperature at approximately 36.5 °C, a warm water circulating heat mat was employed. The respiration and body temperature were continuously monitored during the MRI scans (SA Instruments, Stony Brook, NY, USA). In vivo MRI was performed at 1 day and 7 days post IR treatment. In parallel, the saline-treated group (also referred to as the untreated control) was imaged at the same timepoints, for comparison. We chose 1-day post IR to observe the early effects of treatment, as radiotherapy is known to trigger the release of cytokines in the brain that cause oedema and disruption to the blood–brain barrier [26]. Given that many of the models, particularly the syngeneic GL261, CT-2A, and NPE-IE models, are highly aggressive, the control animals succumb to the tumours 2–3 weeks after implantation, restricting the window in which radiotherapy and MRI can be performed. We therefore chose 7 days post IR as the latest timepoint for MRI to determine whether the changes induced by radiation were maintained and measurable with radiomics.

MRI was performed on a 9.4T BioSpec scanner (Bruker BioSpin GmbH, Ettlingen, Germany) equipped with a rat brain array receiver. Data were acquired with Paravision 7.0 (Bruker BioSpin GmbH, Ettlingen, Germany). All MRI protocols (Table S1) were set to cover the full tumour volume. A T1-weighted MRI protocol was followed for the morphological assessment of tumour volumes. The parameters included the following: TE =14.7 ms, TR = 1550 ms, 30 slices, in-plane resolution (145 × 145) µm^2^, slice thickness of 400 µm, and scan time of 2 min 30 s. For the ADC measurement, a respiration-triggered echo-planar diffusion trace protocol was acquired with the following parameters: TE = 21 ms, TR = 5000 ms, two segments, 6 directions, b-value = 1000 s/mm^2^, gradient duration 3.5 ms, gradient separation 10.5 ms, in-plane spatial resolution (156 × 156) µm^2^, slice thickness 400 µm, scan time = 8 min. Perfusion images were acquired following an ASL-EPI protocol as follows: TE = 9 ms, TR = 4000 ms, 2 segments, in-plane spatial resolution (175 × 175) μm^2^, slice thickness 1 mm, 45 dynamics, and scan time 12 min; cerebral blood flow (CBF) maps were subsequently generated according to the relevant literature [27,28]. Due to strong B_0_ field inhomogeneity near the surgery site, two of the perfusion MRI images (one from a GL261 mouse and one from a CT-2A mouse, both at 1 day post treatment), were excluded from the analysis. MRI images were converted in nifti format. The intensity and resolution dynamic range of original images was preserved, in agreement with the image biomarker standardisation initiative (IBSI) guidelines for radiomic studies. Regarding image post-processing, neither anti-aliasing nor noise suppression were applied. For distortion correction, the echo-planar data were collected with reversed phase-encoding blips, resulting in pairs of images with distortions going in opposite directions. From these pairs, the susceptibility-induced off-resonance field was estimated [29] using FSL [30], and the two images were combined into a single corrected one. Manual tumour segmentation was performed using ITK-SNAP [31] software, version 3.8.0. Normalisation was applied by scaling the images to a range of 0 to 1 to make the images more comparable across different datasets and studies. Tumour radiomic features were then extracted from the ADC and CBF maps using pyradiomics [32]. Three categories of features were derived: shape, and first- and second-order radiomics. Shape features describe the morphological characteristics of segmented tumours, while first-order features include measurements of voxel values and distribution of intensities within tumours. Second-order features, often labelled as texture features, provide information about the relationships between neighbouring voxels within tumours. Second-order features include grey level co-occurrence matrix (GLCM), grey level size zone matrix (GLSZM), grey level run length matrix (GLRLM), neighbourhood grey-tone difference matrix (NGTDM), and grey level dependence matrix (GLDM) features. Additionally, wavelet and Laplacian of Gaussian (LoG) filtered features were extracted (Table S2).

### 2.5. Histopathology

At the end of the MRI experiment, the mice were euthanised using 20 mg pentobarbitone sodium and perfused through the heart with PBS, followed by 4% paraformaldehyde (PFA) in PBS. The brains were removed, stored at 4 °C in 4% PFA, and subjected to FFPE (formalin-fixed paraffin-embedded) processing. Sections of 5 μm thickness were prepared using the HistoCore Multicut microtome (Leica Biosystems, Nußloch, Germany) and mounted on Superfrost Plus Adhesion Microscope slides (J1800AMNZ, Epredia, Portsmouth, NH, USA). For H&E staining, the sections were dewaxed, gradually rehydrated, stained with haematoxylin M (HEMM-OT, Biognost, Zagreb, Croatia) and eosin Y (152881000, Thermo Scientific, Waltham, MA, USA), and then dehydrated.

For immunostaining, the sections were dewaxed, rehydrated, and blocked with 1% H_2_O_2_ (23622.298, VWR, Radnor, PA, USA) for 30 min. The antigen retrieval was performed by boiling the sections for 20 min in Tris-EDTA (pH 9) containing 0.1% Tween 20 (437082q, VWR, Radnor, PA, USA). Following several PBS washes, the slices were blocked for 20 min at room temperature with 2.5% normal horse serum (MP-7401, Vector laboratories, Burlingame, CA, USA) and then incubated overnight with the anti-CD31 (1:400) primary antibody (77699, Cell Signaling Technology, Danvers, MA, USA) at 4 °C. We used anti-CD31 staining to visualise the vasculature, as CD31 (PECAM-1) is a well-established and reliable marker commonly used in various immunoassays to stain endothelial cells [33]. The following day, the sections were washed and incubated for 1 h with the ImmPRESS (Peroxidase) Polymer Anti-Rabbit IgG Reagent (MP-7401, Vector laboratories, USA). The ImmPACT^®^ DAB Substrate Kit (SK-4105, Vector Laboratories, USA) was used for the staining. The nuclei were counterstained with haematoxylin, and the sections were dehydrated. Slides were then fixed under coverslips using the DPX mountant (06522, Sigma-Aldrich, Burlington, MA, USA). Images were acquired using the Aperio AT2 slide scanner (Leica Microsystems, North Ryde, NSW, Australia) and processed with QuPath (Edinburgh, UK). [34].

The cell-detection algorithm implemented in QuPath v0.4.3 was applied to detect and count the nuclei from the H&E stains. The counts were subsequently normalised to the selected regions of interest (ROIs) chosen within the tumour. For vessel quantification, capillaries were segmented in QuPath using a threshold-based pixel classifier. Vessel area was assessed by calculating the mean surface of detected vessels that covered the tumours. Vessel density was measured by dividing the number of detected vessels by the tumour area.

### 2.6. Patient Data

The University of California San Francisco Preoperative Diffuse Glioma MRI (UCSF-PDGM) retrospective dataset [35] was used in this study. Data collection was performed in accordance with relevant guidelines and regulations, and was approved by the University of California San Francisco institutional review board with a waiver for consent. The dataset population used in our study consisted of 20 adult patients (12 men and 8 women) diagnosed with grade IV diffuse gliomas, confirmed through histopathology. These patients underwent preoperative MRI, initial tumour resection, and genetic testing at a single medical centre from 2015 to 2021 [35]. While prior brain tumour treatment was an exclusion criterion, previous tumour biopsy was permitted. MGMT methylation status was assessed using a methylation-sensitive quantitative PCR assay. Preoperative MRI scans were conducted on a 3.0 tesla scanner (Discovery 750, GE Healthcare, Waukesha, WI, USA) with an 8-channel head coil (Invivo, Gainesville, FL, USA), incorporating pre- and post-contrast T1-weighted images, arterial spin labelling (ASL) perfusion images, and 2D diffusion ADC images. Tumour segmentation focused on two primary compartments: enhancing tumour and non-enhancing/necrotic tumour.

### 2.7. Statistical Analyses

Statistical analysis was performed using GraphPad Prism 8 (GraphPad Software, San Diego, CA, USA). All of the results are presented as means with the corresponding standard deviations (±SD). One-way analysis of variance (ANOVA) was employed for multi-group analysis with Dunnett testing for multiple comparison testing. Power analysis for one-tailed paired-samples *t*-test [36] was performed with alpha set at 0.05. The number of animals per group was determined to match the minimum sample size required to distinguish diffusion and perfusion tumour characteristics from the healthy contralateral side with a statistical power of 80–95%. Histological analyses were performed using Student’s *t*-tests. A *p*-value of <0.05 was taken to represent statistical significance. Data acquisition and analysis was performed in a blinded fashion.

### 2.8. Radiomic Feature Selection and Statistical Evaluation

A supervised statistical-based approach was implemented for feature selection (FS) [37]. The feature selection process employed a two-step approach to identify the most informative and non-redundant radiomic features. Initially, the maximum relevance, minimum redundancy (mRMR) algorithm [38] was applied as a preprocessing step to identify features highly correlated with the GL261, CT-2A, NPE-IE, and GBM96 groups while minimising inter-feature correlations. The F-statistic was used to measure class correlation, while the Pearson correlation coefficient assessed feature-to-feature relationships. Subsequently, the Least Absolute Shrinkage and Selection Operator (LASSO) regularisation algorithm [39] was implemented to further refine the feature set, removing remaining redundancies and enhancing the overall accuracy and interpretability of the feature selection. This combined approach leveraged mRMR’s ability to ensure a diverse set of features while mitigating LASSO’s potential struggle with correlated predictors, resulting in an optimised feature subset for subsequent analysis. All FS techniques were implemented using the Python scikit-learn ML package (version 3.6) [40]. A support vector machine (SVM) was implemented as the primary classification tool to distinguish between the different GB animal models. The SVM classifier was trained using the selected subset of radiomic features which captured characteristics of the tumour’s appearance and texture. The performance of FS and SVM was evaluated and validated by means of confusion matrix analysis, stratified four-fold cross-validation (train/test of 75/25 split ratio) and accuracy measurement (scikit-learn, Python 3.6).

## 3. Results

### 3.1. High Diffusion Coefficients and Low Perfusion Were Found in GL261, CT-2A, and GBM96, but Not in NPE-IE Tumours

**Figure 1:**
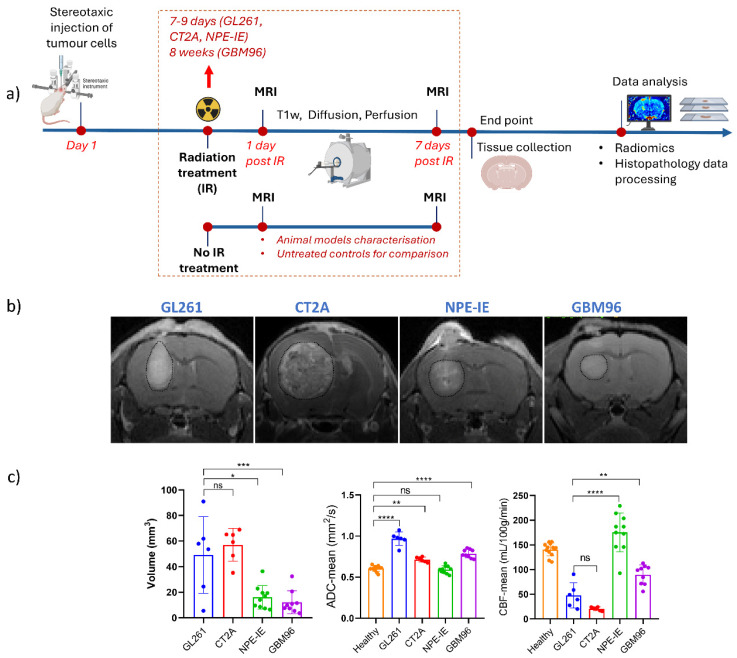
Assessment of tumour volume, diffusion ADC, and perfusion CBF in GL261, CT-2A, NPE-IE, and GBM96 orthotopic tumours. (**a**) Schematic illustration of the experimental design of this study marking the timepoints of cell injection, radiation treatment, MRI scans, and subsequent radiomics and histopathology analysis (figure created with BioRender). (**b**) Representative T1-weighted images highlight the presence of tumours in all four GB models. ADC and CBF measurements were performed on the segmented tumours as indicated by the contoured dotted lines. (**c**) Tumour volume, ADC, and CBF were evaluated in the mouse GB models (mean ± SD, * *p* < 0.05, ** *p* < 0.01, *** *p* < 0.001, **** *p* < 0.0001, one-way ANOVA, comparison to healthy contralateral site, Dunnett correction for multiple comparisons): GL261 (*n* = 6), CT-2A (*n* = 6), NPE-IE (*n* = 10), and GBM96 (*n* = 9).

The experimental design of this study is illustrated in Figure 1a. Tumour morphology, microstructure, and perfusion were assessed in the four orthotopic GB models (Figure 1b) through measurements of tumour volume, ADC, and CBF from the specified regions of interest presented in (Figure 1b). CT-2A showed the highest level of tumour volume growth. Most animal models demonstrated significantly higher ADC values when compared to the healthy contralateral side with GL261 displaying the highest ADC among them. Interestingly, maintained ADC and superior perfusion values were noticed in the NPE-IE model. GBM96 tumours showed slightly lower CBF in comparison, whereas CT-2A and GL261 had the lowest perfusion overall.

### 3.2. Diffusion and Perfusion Radiomic Profiling Demonstrates Notable Differences Among Orthotopic Glioma Tumours

**Figure 2:**
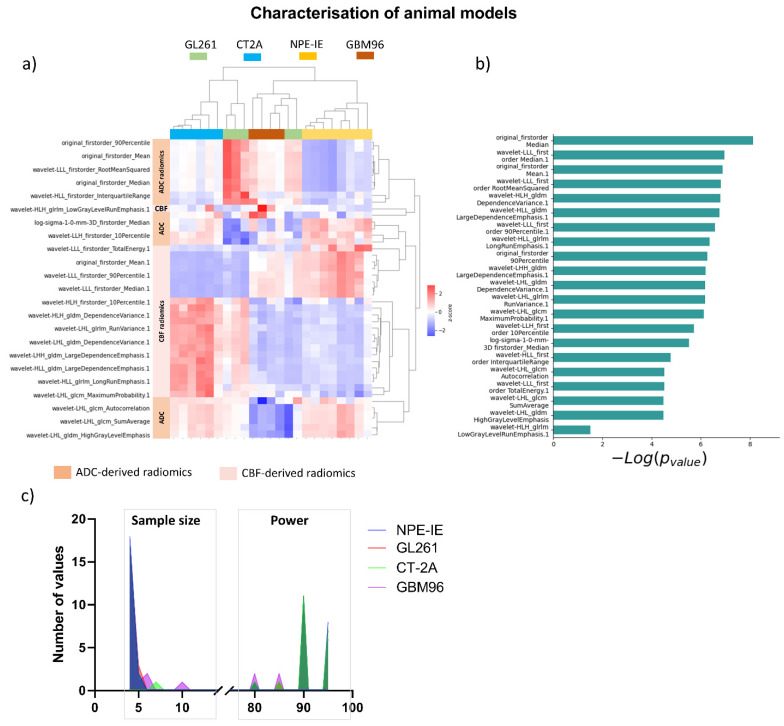
Radiomics distinguish between GL261, CT-2A, NPE-IE, and GBM96 models with high statistical significance. (**a**) Dendrogram and heat map of hierarchical clustering analysis can distinguish the four animal models using the selected radiomic features. (**b**) Ranking of selected features according to their *p*-value to indicate the statistical significance to the target variable. (**c**) Frequency distribution plot illustrating the number of animals needed to attain statistical power between 80 and 95%. We note that *n* = 4 has the highest number of counts for each of the murine models, with very few features from the CT-2A and GBM96 model requiring 5 to 6 mice.

The most relevant radiomic features selected using the mRMR algorithm and LASSO regularisation were extracted from CBF and ADC maps to effectively characterise each animal model, with the aim of gaining a more detailed understanding of tumour characteristics through radiomic profiling. The dendrogram and heat map generated from the hierarchical clustering of selected radiomics indicated that NPE-IE and CT-2A display unique traits associated with specific CBF- and ADC-derived radiomics (Figure 2a), forming two distinct clusters, when compared with the other GB models. There were resemblances observed among GBM96, NPE-IE, and certain GL261 tumours in terms of wavelet-transformed GLDM_LargeDependenceEmphasis, GLDM_DependenceVariance, and GLRLM_RunVariance CBF radiomics. GBM96 revealed similitudes to NPE-IE regarding the mean and range values of CBF. GBM96 showed noticeable differences from the other GB models in relation to ADC wavelet transformed features of LHL_GLCM_SumAverage, LHL_GLCM_Autocorrelation, and LHL_GLDM_HighGrayLevel_Emphasis. Furthermore, the four GB models were distinctly different according to the first-order values of average, median, and 90th percentile ADC.

To evaluate the statistical significance of the selected features in differentiating among the classes, we have ranked them according to their *p*-value (−log(*p*-value)) (Figure 2b). The minimum sample size required to detect significant changes between the different animal models indicated that most of the selected features necessitated a relatively small sample size (*n* = 4–5) and a statistical power ranging from 80% to 95% (Figure 2c).

### 3.3. IR Therapy Leads to Increased ADC and Improved CBF in GL261 and CT-2A Tumours

**Figure 3:**
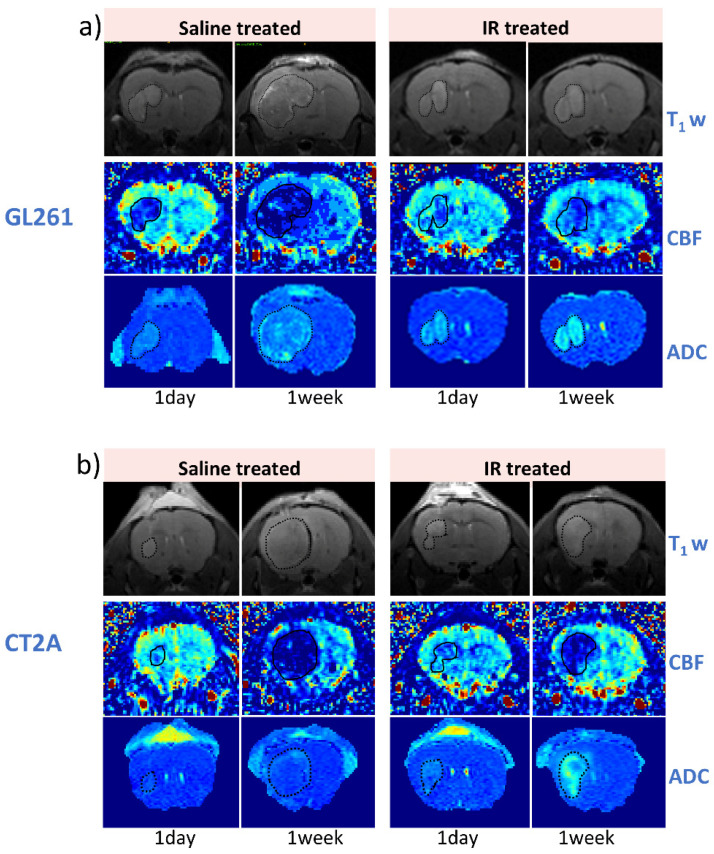

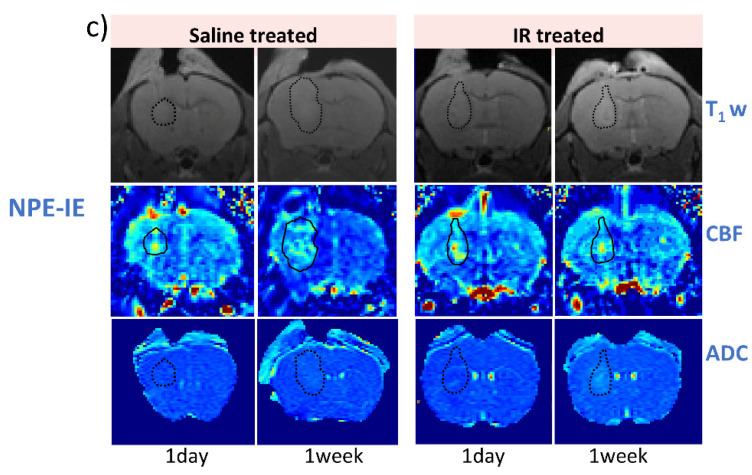
Longitudinal evaluation of tumour response to saline– and IR treatment through MR imaging. Representative T1-weighted, CBF, and ADC mapping of (**a**) GL261, (**b**) CT-2A, and (**c**) NPE-IE models. Segmentation of tumours is indicated by the contoured dotted lines. Darker contrasts represent hypoperfused tumours in the CBF maps, and restricted diffusion in the ADC maps. Brighter contrast represents higher perfusion values in the CBF maps, and less restricted diffusion and/or oedema in the ADC maps.

Figure 3 illustrates representative examples of T1-weighted, CBF, and ADC maps collected at 1 day and 7 days following saline and IR treatment for the GL261 (Figure 3a), CT-2A (Figure 3b), and NPE-IE models (Figure 3c), respectively. The change in tumour ADC and CBF was very distinguishable, particularly at the 7-day timepoint.

**Figure 4:**
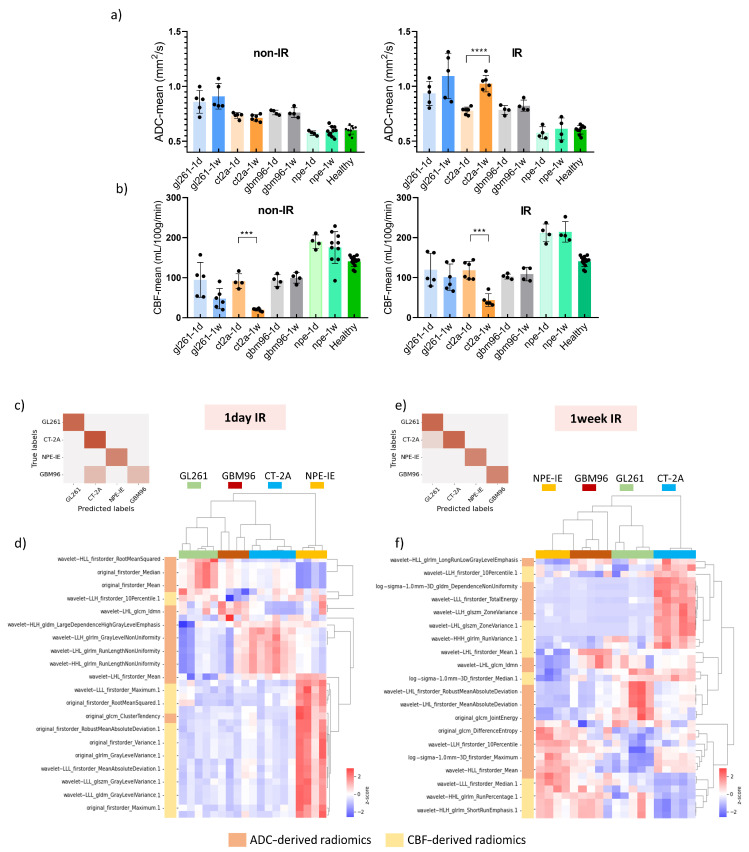
Quantification of ADC and CBF changes following irradiation. (**a**) ADC changes 1 day and 7 days following saline and IR treatment. (**b**) CBF changes 1 day and 7 days following saline and IR treatment (mean ± SD, *** *p* < 0.001, **** *p* < 0.0001, one-way ANOVA, comparison between 1-day/7-day groups, Dunnett correction for multiple comparisons). The black dots represent individual data points corresponding to each of the *n* animals in the study, namely: GL261 (*n* = 5 to 6 per treatment group, per MRI timepoint), CT-2A (*n* = 5 to 6 per treatment group, per MRI timepoint), NPE-IE (*n* = 10 for 1-week saline-treated group, *n* = 4 for remaining groups), GBM96 (*n* = 4 per treatment group, per MRI timepoint). Confusion matrices build from the mouse GB models at (**c**) 1 day and (**e**) 7 days post IR. Dendrogram and heat map of hierarchical clustering analysis performed at (**d**) 1 day and (**f**) at 7 days post IR treatment.

MRI indicated reduced tumour growth in the IR group compared to saline-treated controls across all animal models. Diffusion MRI showed increased ADC values at 7 days post IR, particularly in the GL261 and CT-2A models (Figure 4a). The saline-treated group initially presented restricted diffusion followed by a moderate increase mid-stage. Tumour CBF significantly increased 1 day post IR in GL261 and CT-2A, as opposed to controls, which showed drastic CBF reduction (Figure 4b).

The GBM96 model showed similar trends, with a lower intensity of change. Given that GBM96 tumours develop at a slower rate than the other, more aggressive, syngeneic models, it was expected that the impact of irradiation would be more pronounced on rapidly growing GL261, CT-2A, and NPE-IE tumours.

Although tumour CBF presented a slight decline 1 week post IR, perfusion remained substantially higher in the NPE-IE model compared to GL261, CT-2A, and GBM96.

### 3.4. Radiomic Descriptors of MRI Heterogeneity Show Specific Changes 1 Day and 7 Days Post IR

We further examined the capability of ADC and CBF radiomics to generate distinct signatures that could serve as indicators of treatment response following irradiation. Using the mRMR FS algorithm, radiomic features were selected based on their relevance to the target variable. LASSO regularisation further identified the most optimal subset of features for further classifying the GB groups according to their treatment response. A total of twenty metrics (features), predominantly from wavelet-transformed images, were obtained. The dendrogram and heat map resulting from the hierarchical clustering of selected features revealed treatment responses distinctively associated with each of the four GB models. The accuracy of the here proposed classification was evaluated, at 1-day and 7-days post-IR timepoints, using a confusion matrix build for the GL261, CT-2A, NPE-IE, and GBM96 groups.

One day post IR: In general, the data demonstrated distinct separation between the classes (Figure 4c). Specifically, the GL261, CT-2A, and NPE-IE groups were 100% correctly predicted (accuracy 100%), whilst few misclassified cases were found in the GBM96 group (accuracy 89%). Inspecting the dendrogram in Figure 4d, we noted some similarities between GBM96–GL261 and GBM96–CT-2A one day post IR. Specifically, the GBM96 and CT-2A models exhibited comparable changes in mean, median, and root mean squared of the ADC values. Certain GBM96 tumours showed similarities to GL261, while others to CT-2A in terms of ADC wavelet-transformed GLRLM_GrayLevelNonUniformity (GLNU) radiomics, indicating comparable image heterogeneity (variability in size and grey intensity levels) of GMB96 akin to either CT-2A or GL261. NPE-IE provided a distinct profile response, mainly determined by the CBF derived radiomic features. This difference may be attributed to the elevated blood perfusion observed in this model prior to—and following irradiation (Figure 4b).

One-week post IR: the data showed good separation between the four classes as illustrated in Figure 4e. The CT-2A, NPE-IE, and GBM96 groups were 100% correctly predicted (accuracy 100%), whilst one misclassified case was obtained in the GL261 group (accuracy 94%). The dendrogram in Figure 4f shows that the CT-2A model displayed distinct responses compared to the other GB models, as indicated by alterations in several ADC and CBF wavelet-transformed radiomics, which may reflect increased tumour heterogeneity (GLRLM_GrayLevelNonUniformity—GLNU), larger tumour volume growth (firstorder_TotalEnergy), and greater variability in the size of homogeneous intra-tumoral zones (GLSZM_ZoneVariance, GLSZM_RunVariance) post IR. NPE-IE and GBM96 exhibited a degree of similarity in the following radiomics, indicating comparable image homogeneity after irradiation: GLRLM_differenceEntropy, GLRLM_RunPercentage, and GLRLM_ShortRunEmphasis.

**Figure 5:**
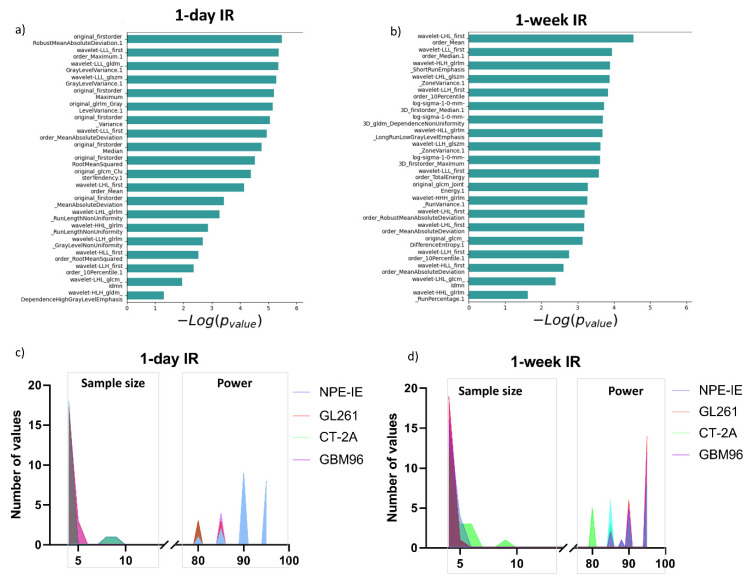
Ranking of selected radiomic features according to their significance to differentiate treatment responses 1 day and 7 days post IR. (**a**) Ranking of selected features according to their *p*-value to indicate the statistical significance to classify (**a**) 1-day and (**b**) 7-days post-irradiation responses in the four animal models. (**c**,**d**) Frequency distribution plot illustrating the sample size needed to achieve statistical power between 80 and 95% for 1 day and 7 days post irradiation. We note that *n* = 4 has the highest number of counts for each of the models, with very few features (derived from CT-2A and GBM96) requiring an *n* number of 5 to 6.

To assess whether the selected features are statistically significant in distinguishing changes at the 1-day and 7-days post-IR timepoints, we ranked the selected features based on their *p*-value (−log(*p*-value)) as illustrated in Figure 5a,b. The minimum sample size required to identify significant alterations in the treatments groups revealed that most of the selected features require a relatively small sample size (*n*  =  4–6) and 80% to 95% statistical power (Figure 5c).

### 3.5. High Tumour Cellularity Is Present in All Orthotopic Tumours Along with Dilated Vessels in GL261 and CT-2A Tumours

**Figure 6:**
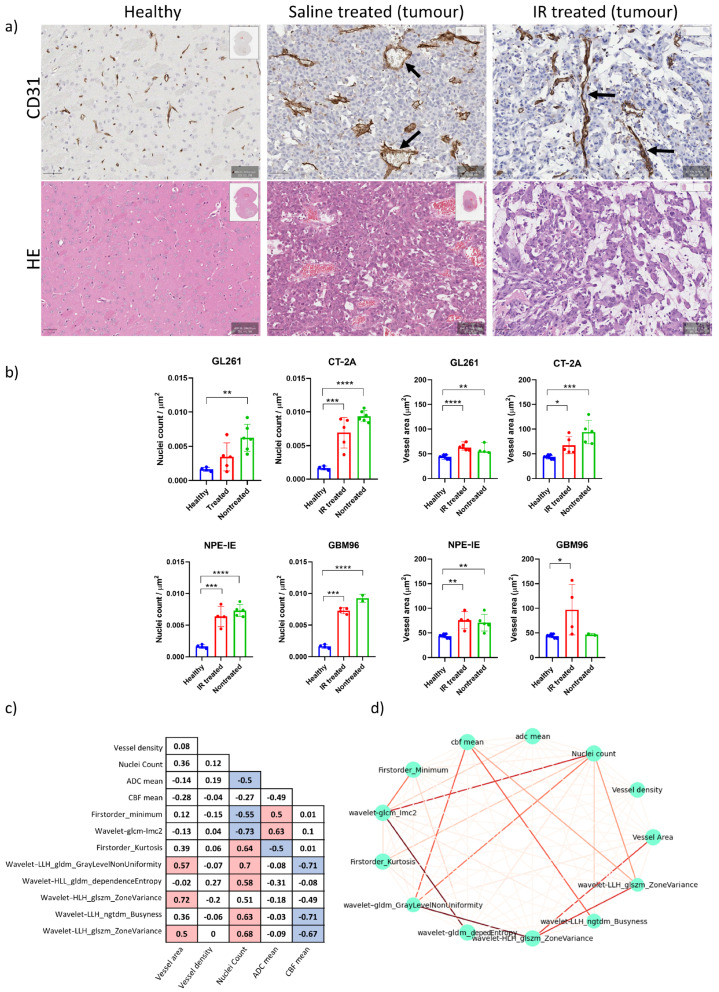
Histopathology reveals high tumour cellularity and dilated vessels in orthotopic GB tumours. (**a**) Representative magnified histology micrographs are displayed showing an example of increased nuclear count (hematoxylin and eosin, scale bar 50 µm) and dilated vessels (CD31, scale bar 50 µm) in the saline-treated mice, as opposed to IR-treated and healthy cases. We note the prominent dilatation and wall thickening of vessels in tumours (dark arrows) as well as visibly reduced vessel branching compared to the healthy contralateral side. (**b**) Quantification of nuclear count and vessel area changes in saline- and IR-treated mice, in comparison to healthy mice (mean ± SD, * *p* < 0.05, ** *p* < 0.01, *** *p* < 0.001, **** *p* < 0.0001, *n* = 4–5 per group, *t*-test). (**c**) Correlation matrix of radiomic features having highest correlation to histopathology. (**d**) Correlation network build from radiomics and histopathology markers of nuclear count, vessel area, and vessel density illustrating the strength of correlations (thicker lines represent stronger correlations).

Magnified histology micrographs in Figure 6a show an example of H&E and CD31 staining highlighting differences in radiation treatment response between the saline-treated and IR-treated group.

Histopathological analysis of H&E stains revealed increased nuclear counts across all untreated controls, confirming the increased cellularity of these tumours (Figure 6b). CD31 analysis showed chaotic blood vessel network distribution in saline-treated control groups, particularly in the GL261 and CT-2A mice, which exhibited enlarged vessel diameters. Following IR treatment, decreased vessel dilation was observed in the CT-2A mice, as well as reduced nuclei counts across all animal models. No notable change in vessel diameter was observed post IR in the GL261, NPE, and GBM96 mice (Figure 6b).

### 3.6. Radiomics Yielded Higher Correlations with Histopathology than ADC and CBF Alone

**Figure 7:**
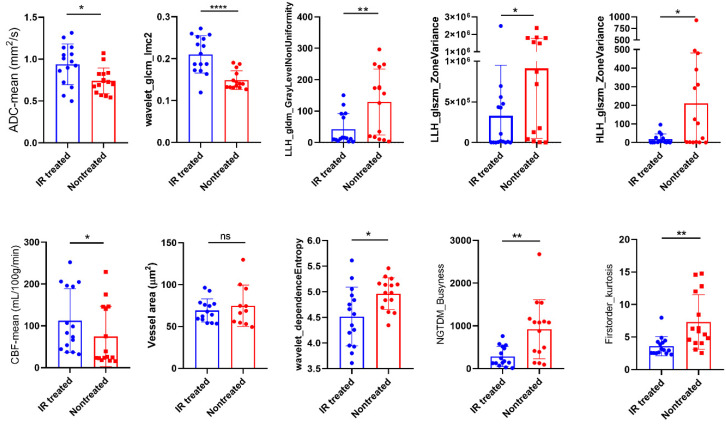
Radiomics highlight differences between the IR-treated and saline-treated groups. Comparison between saline- and IR-treated responses of selected radiomics along with the ADC and CBF responses (mean ± SD, * *p* < 0.05, ** *p* < 0.01, **** *p* < 0.0001, *n* = 4 per group, *t*-test). The radiomic features showing highest correlations with histopathological markers of nuclei count and vessel area were used for this analysis.

We examined the features that most effectively characterised the 1-week post saline- and post IR treatment across all models to identify those that correlate strongly with histopathologic parameters related to cellular density, vessel dilation and density, CBF mean, and ADC mean. The correlation matrix displayed in Figure 6c illustrates the interdependencies among these wavelet-transformed MRI radiomic features. Seven features showed strong correlations with coefficients ranging from 0.6 to 0.73. The strength of correlations is illustrated in the correlation network of Figure 6d, where thicker lines represent stronger correlations. The wavelet_GLCM_Imc2, LLH_GLDM_GLNU, firtsorder_Kurtosis, and LLH_ GLSZM_ZoneVariance exhibited significant correlation with nuclei counts, whereas HLH_GLSZM_ZoneVariance was found to be correlated with vessel area. Furthermore, LLH_ GLDM_GLNU, LLH_NGTDM_Busyness, and LLH_GLSZM_ZoneVariance were associated with mean CBF, while wavelet_glcm_Imc2 correlated with mean ADC. These findings suggest that the identified features create a radiomic profile that is closely linked to the response to IR treatment of these tumour models in terms of tumour cellularity and vessel dilation. Moreover, features such as wavelet_GLCM_Imc2, LLH_ GLDM_GLNU, LLH_NGTDM_Busyness, and firstorder_Kurtosis displayed a more pronounced difference between treated and nontreated mice compared to ADC and CBF alone (Figure 7), while the others exhibited comparable results to both ADC and CBF metrics. Differentiating these characteristics using radiomics is of high value in preclinical research for assessing treatment responses over time, potentially eliminating (or reducing) the necessity for histopathology.

### 3.7. Mouse Orthotopic Tumours Share Similarities with Central Non-Enhancing Patient Tumours

**Figure 8:**
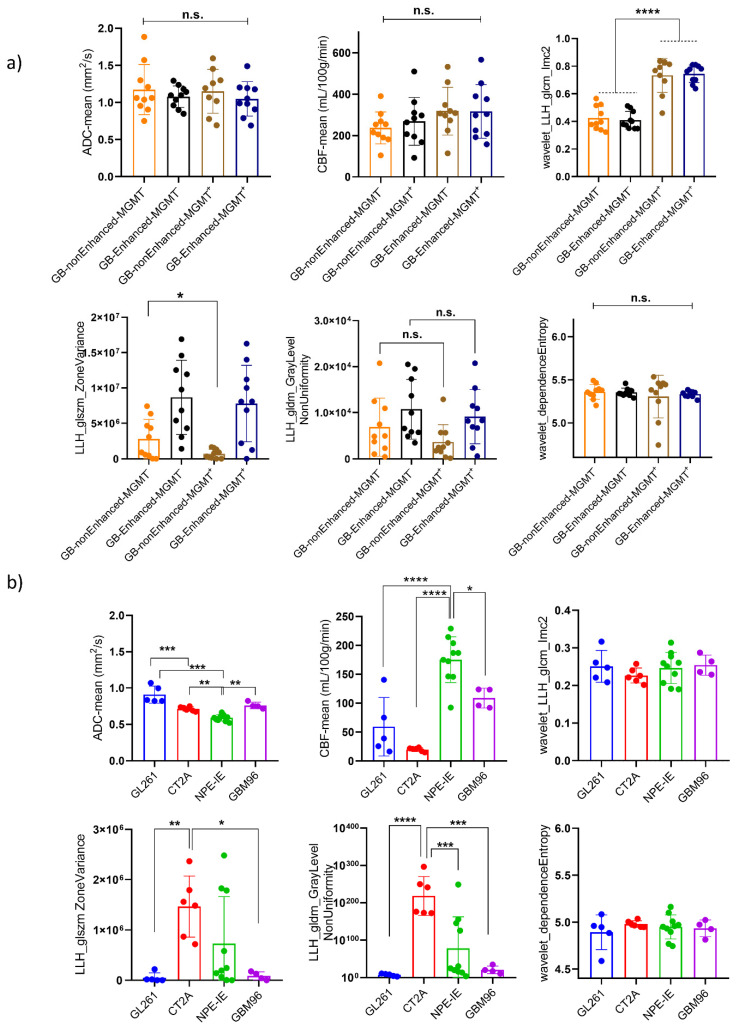
Comparison of mouse orthotopic GB tumours to GB patients. Comparison between (**a**) non-enhanced and enhanced tumour regions of MGMT (−) and MGMT (+) patients and (**b**) between the four animal models according to the selected radiomic features as well as ADC and CBF values. Mouse orthotopic tumours share similarities and several differences with central non-enhancing patient GBs (mean ± SD, * *p* < 0.05, ** *p* < 0.01, *** *p* < 0.001, **** *p* < 0.0001, one-way ANOVA, Dunnett correction for multiple comparisons): GL261 (*n* = 5), CT-2A (*n* = 6), NPE-IE (*n* = 10), GBM96 (*n* = 4), MGMT(+) (*n* = 10), MGMT(−) (*n* = 10).

When comparing them to the patients’ MRI data, we observed that only the NPE-IE model presented comparable CBF values to the non-enhanced central tumours in GB patients (Figure 8). The other tumour models revealed significantly lower perfusion values in comparison to the patient dataset. The ADC values of all orthotopic models were significantly lower than those of the patient dataset. This is primarily because orthotopic tumours are known to be highly solid, with very dense cell distribution, limiting the diffusion of water within them.

We further examined the behaviour of the wavelet-transformed radiomic set that exhibited stronger correlations with histopathological markers of vessel area and tumour cellularity in the orthotopic models when applied to patient data.

Overall, the patients showed more heterogeneous presenting tumours, with greater variability in the size and grey intensity levels of intra-tumoral pixel distribution. This was reflected by the markedly higher values of HLL_GLDM_dependenceEntropy, HLH_GLSZM_ZoneVariance, and LLH_GLDM_GrayLevelNonUniformity radiomic features. An interesting observation was that the variability in the size of homogeneous intra-tumoral zones in all orthotopic models closely resembled that of the non-enhanced central tumours of GB patients. This was indicated by the similarly low values of LLH_GLSZM_ZoneVariance seen in both mouse and GB patients.

Furthermore, we have observed a notable distinction between MGMT methylated, MGMT(−), and MGMT unmethylated, MGM(+), patient tumours in terms of the wavelet_GLCM_Imc2 feature. Namely, MGMT(+) patients exhibited a more complex texture pattern of wavelet transformed ADC maps, characterised by a wider range of intensity relationships among neighbouring pixels, in contrast to the MGMT(−) patients.

## 4. Discussion

Orthotopic models of GB are essential tools for the preclinical evaluation of therapies. They can mimic features of tumour growth and invasion and have the ability to replicate certain traits of human GB [23]. However, to date, the results obtained in preclinical models of GB have failed to translate in the clinical setting. Introducing novel methods for detailed non-invasive characterisation of these models could capture more intricate details of responses to treatments, thereby facilitating the discovery of new diagnostic biomarkers that may help assess the efficacy of therapies more robustly.

In this study, we developed a thorough radiomic phenotyping analysis of four mouse orthotopic GB models by utilising a combination of quantitative MRI protocols to assess tumour characteristic changes following irradiation. Of the three syngeneic models, two were chemically induced (CT-2A and GL261) and one (NPE-IE) represented a spontaneous model of GB. The fourth was a xenograft model using a human GB primary cell line which allowed us to examine the effects of IR treatment on real patient tumours. We showed that diffusion and perfusion radiomics provide information about tumour cellularity and degree of tumour vascularization that are not evident on standard MRI visual inspection. The assessment of MRI textural features and spatial correlation of grey levels in ADC and CBF maps, in conjunction with analysis of coarseness and regularity of local variations in MR image intensity, offered further insights of tumour spatial heterogeneity responses post IR treatment. The histopathological examination provided further clarification on the interpretation of these selected radiomic features by showing high correlation with nuclear counts and vessel appearance/distribution.

CT-2A tumours are recognised for their haemorrhagic and infiltrative characteristics, marked by high cell density, rapid growth, and expansion [41]. Our results indicated that, both with and without IR treatment, the CT-2A model exhibited increased heterogeneity in ADC values (high GLNU values) and greater blood perfusion heterogeneity (high values of LHL_GLSZM_ZoneVariance) when compared to the other models, with histopathologic correlations of 0.7 and above. CD31 staining revealed a disordered network of blood vessels, characterised by tortuous, enlarged vessels with profoundly reduced branching and increased vessel diameter. This could lead to irregular blood perfusion and vascular leakage, which, in conjunction with the high cell density noted in this model, might account for the low perfusion values seen in CT-2A. Given that CT-2A showed the highest tumour growth in this study, the set of radiomic features showing elevated GLNU radiomic values, high total energy, and high LHL_ZoneVariance could serve as a diagnostic imaging biomarker for identifying more advanced orthotopic tumours.

The NPE-IE cells are known for their ability to form highly aggressive tumours with increased tumour growth [18]. This increased expansion likely requires an extensive blood supply to support rapid tumour expansion. Additionally, their similarities to the mesenchymal subtype of human GB, which is known for its elevated angiogenic signatures relative to other subtypes, may explain the high perfusion values noted in the NPE-IE tumours, with values nearly reaching those seen in central non-enhancing tumours of GB patients.

The GBM96 and GL261 tumours presented a higher degree of uniformity in tumour texture and finer image coarseness post IR when compared to CT-2A. This may be due to their comparable CBF values observed after IR, along with a diminished response in vessel area reduction post IR in contrast to CT-2A. Similarities were also noted between the NPE-IE and GBM96 models in terms of image homogeneity following irradiation. These are likely attributed to the high prevalence of elevated CBF/low ADC values in both models, leading to comparable intra-tumoral pixel intensity levels and uniformity in grey level size distribution.

We therefore show that radiomics analysis of ADC and CBF metrics can effectively capture changes in microstructural, textural, and vascular heterogeneity of murine tumours both prior to and following IR treatment. Specifically, our findings indicated that GL261 and CT-2A exhibit characteristics of hypoperfused and hypercellular tumours, whereas NPE-IEs are hypervascularised and hypercellular. This suggests that ADC and CBF radiomics could facilitate the identification of distinct regions within the same tumour with varying combinations of hyper- or hypo-perfusion, cellularity, and varying image texture levels. This holds significant promise in a clinical scenario for the evaluation of changes in physiology and tumour habitat composition post IR [6], potentially enabling the identification of treatment-resistant tumours prior to therapy which are likely to relapse after standard radiation treatment [42]. The ability to differentiate between hypercellular tumours and assess microstructural and perfusion heterogeneity levels is crucial for treatment planning, survival prediction, and risk stratification [8]. Furthermore, this may aid in the segmentation of radiotherapy target volumes [43] and enhance the assessment and prediction of treatment responses, ultimately contributing to more personalised and effective treatment strategies for GB patients.

A general observation was that the diffusion values of orthotopic tumours were significantly lower than those of GB patients. This indicates that the tumours in the mouse models are highly solid, with denser cellularity in comparison to patients.

Overall, the radiomics analysis demonstrated clear differences in image heterogeneity between patient and mouse orthotopic tumours. GB patient tumours exhibited greater heterogeneity in terms of several wavelet transformed ADC features, which may be explained by the greater diversity in cell populations and/or microenvironments at molecular [44], microscopic [45], and macroscopic [46] levels found in patients as opposed to animal models.

Interestingly, we have observed comparable lower values of ADC wavelet transformed LLH_GLSZM_ZoneVariance in both orthotopic and central non-enhancing patient tumours. Lower values of LLH_GLSZM_ZoneVariance indicate greater homogeneity in the size of areas with similar intensity within the tumour. The central non-enhancing GB tumours do contain solid tissue composition with substantial areas of necrosis [47] and lower perfusion, which may reflect a more homogeneous distribution of pixel intensity, akin to that observed in mouse orthotopic tumours. This might also explain the differences in LLH_GLSZM_zoneVariance and LLH_GLDM_GLNU observed between the enhancing and non-enhancing tumours of these patients.

In addition, the differences in image texture and complexity observed between the two groups of GB patients (MGMT(+) and MGMT(−)) are noteworthy, as identifying MGMT promoter methylation status non-invasively through radiomics would be of great importance for GB patients in evaluating their response to chemotherapy and for the improvement of their prognosis [48]. Given the role of MGMT in the repair of damaged DNA, the lack of repair enhances genomic instability, which could manifest as distinct image textures and complexity in MRI scans. MGMT methylation may also influence the cellular makeup of the tumour [49], affecting the distribution of necrotic areas and enhancing/non-enhancing regions.

### Challenges and Limitations

We acknowledge that there have been difficulties translating findings from murine models of glioma to clinical benefits for patients with GB. The NPE-IE model, a spontaneous GB model, had specifically been developed to address some of these limitations [18]. Our study confirms this by demonstrating that the NPE-IE model shares similarities with human GBs regarding tumour perfusion metrics and intra-tumoral signal intensity distribution patterns, making it unique among the herein studied murine models. 

Currently, there are no animal alternatives able to adequately replicate the complex features of brain tumours, particularly in relation to the blood–brain barrier and the tumour immune microenvironment. The application of our radiomic framework in future pre-clinical studies will help with the selection of animal models that more accurately reflect human tumours. Refining animal model selection will enhance the translation of pre-clinical studies, helping to accelerate the development of more effective treatments, ultimately improving the management of GB patients.

Challenges continue to exist in the standardisation and biological validation of radiomic features across different studies and imaging protocols, making this an area of ongoing development. International harmonisation of imaging protocols and reporting is crucial for advancing radiomics research and its clinical application. Standardised guidelines for image acquisition, reconstruction parameters, and post-processing techniques can minimise variability and ensure consistency across multicentre studies. Initiatives like those by the Food and Drug Administration (FDA) and IBSI [50] have demonstrated the importance of such efforts in improving feature reproducibility [51].

## 5. Conclusions

We showed that MRI radiomics can extract important diffusion and perfusion information about the entire tumour’s volume, vascularization, and cellularity, that may be missed by biopsies and human-eye-evaluated MRIs. As the field advances, it has the potential to greatly improve preclinical cancer research, serving as a valuable virtual biopsy alternative. The quantitative MRI techniques outlined here are readily available on standard clinical scanners, enhancing the potential of translating these preclinical radiomic findings into clinical practice.

## Data Availability

The data presented in this study may be distributed upon request.

## Supplementary Materials

The following supporting information can be downloaded at: https://www.mdpi.com/article/10.3390/cancers17081258/s1, Table S1: Glossary with the MRI protocols and computed parameters used in this study; Table S2: Summary of radiomic features and feature selection methods used in this study.
