# Supplementary material for: Radiomic Profiling of Orthotopic Mouse Models of Glioblastoma Reveals Histopathological Correlations Associated with Tumour Response to Ionising Radiation
Source: Cancers (Basel). 2025 Apr 8;17(8):1258. doi: 10.3390/cancers17081258 (PMC12025692; doi:10.3390/cancers17081258)
Supplement: Supplementary file 1 [file cancers-17-01258-s001.zip › cancers-3481294-supplementary.pdf]

Radiomic profiling of orthotopic mouse models of glioblastoma reveals histopathological correlations associated with tumour response to ionizing radiation

Nicoleta Baxan [1,2]*, Richard Perryman [1], Maria Chatziathanasiadou [1] and Nelofer Syed [1]*

[1]John Fulcher Neuro-Oncology Laboratory, Department of Brain Sciences, Faculty of Medicine, Imperial College London, London W12 0NN, UK

[2]Biological Imaging Centre, Hammersmith Campus, Imperial College London, London W12 0NN, UK

*Authors to whom correspondence should be addressed

Supplementary material

**Diffusion MRI**	Echo-planar MRI diffusion trace protocol	Apparent diffusion coefficient (ADC)	b-value: setting controlling the scan sensitivity to water movement in tissues
**Perfusion MRI**	Arterial Spin Labelling (ASL)	Cerebral blood flow (CBF)	

Table S1: Glossary with the MRI protocols and computed parameters used in this study.

	First Order Statistics	*Texture Features*				
**Radiomic features (*extracted using pyradiomics*)**	Energy<br>Entropy<br>Mean<br>Median<br>Standard Deviation<br>Skewness<br>Kurtosis	Gray Level Co-occurrence Matrix (GLCM)	Gray Level Run Length Matrix (GLRLM)	Gray Level Size Zone Matrix (GLSZM)	Neighbouring Gray Tone Difference Matrix (NGTDM)	Gray Level Dependence Matrix (GLDM)
**Filters**	Wavelet		Laplacian of Gaussian (LoG)			
**Feature selection**	Minimum Redundancy Maximum Relevance (mRMR)		Least Absolute Shrinkage and Selection Operator (LASSO)			

Table S2: Summary of radiomic features and feature selection methods used in this study
